# Testing the Isotropic Cauchy Hypothesis

**DOI:** 10.3390/e26121084

**Published:** 2024-12-11

**Authors:** Jihad Fahs, Ibrahim Abou-Faycal, Ibrahim Issa

**Affiliations:** 1 Department of Electrical and Computer Engineering, American University of Beirut, P.O. Box 11-0236, Beirut 1107 2020, Lebanon; ibrahim.abou-faycal@aub.edu.lb (I.A.-F.); ibrahim.issa@aub.edu.lb (I.I.); 2 Center for Advanced Mathematical Sciences, American University of Beirut, P.O. Box 11-0236, Beirut 1107 2020, Lebanon

**Keywords:** heavy-tailed, alpha-stable, Cauchy, hypothesis testing, Bayesian detection, Neyman–Pearson, isotropic, correlated

## Abstract

The isotropic Cauchy distribution is a member of the central α-stable family that plays a role in the set of heavy-tailed distributions similar to that of the Gaussian density among finite second-moment laws. Given a sequence of *n* observations, we are interested in characterizing the performance of Likelihood Ratio Tests, where two hypotheses are plausible for the observed quantities: either isotropic Cauchy or isotropic Gaussian. Under various setups, we show that the probability of error of such detectors is not always exponentially decaying with *n*, with the leading term in the exponent shown to be logarithmic instead, and we determine the constants in that leading term. Perhaps surprisingly, the optimal Bayesian probabilities of error are found to exhibit different asymptotic behaviors.

## 1. Introduction

Detecting the underlying sampling distribution of an empirical set of *n* observations is a challenging and fundamental question in statistics. It falls under the family of hypothesis testing problems whenever deciding on a possibility among a given number of choices. In the binary scenario (*p* vs. *q*), it is well known that Likelihood Ratio Test (LRT) detectors are optimal for multiple setups. Under a Neyman–Pearson criterion or under a Bayesian risk formulation, the performance of such detectors is expected to be exponentially decaying with *n*, with the exponent being a Kullback–Leibler (KL) distance between pertinent laws. This is the case in a discrete scenario [1] (Th. 11.8.3 & Th. 11.9.1), where a well-known proof is based on the method of types. Extensions to continuous setups do exist under certain conditions [2,3].

One interesting quest is to analyze this decision problem while testing for:

1. The Cauchy distribution: representative of the family of “heavy-tailed” α-stable distributions versus;
2. The Gaussian distribution: a central model among finite-variance probability laws.

Note that both models are limiting distributions of Independent and Identically Distributed (IID) sums of Random Variables (RV)s by the results of the generalized Central-Limit Theorem [4] and are therefore supported by strong theoretical evidence whenever the observations are the result of the accumulation of a large number of random independent events. While the Gaussian model has been dominant in the literature, with both theoretical and empirical justifications, recent studies suggested the non-suitability of the Gaussian models and proposed heavy-tailed ones including the α-stable family in a multitude of scenarios: interference modeling in digital communication [5,6], radar clutter data [7], internet traffic modeling [8], and financial data [9] among many other examples that can be found in [10,11]. In the multivariate case, random feature models are used to better understand several complex behaviors of neural networks [12,13,14]. In [15], the authors consider the problem of classification features in high dimensions by assuming a heavy-tailed data model according to a superstatistical construction [16]: the feature vector X is assumed to be distributed according to ZG, where G is multivariate Gaussian with IID components, and *Z* is a positive RV. This defines a dependent statistical model for the observed data and has been found suitable in many other instances [17,18].

Among the isotropic (we will use “circular” interchangeably with “isotropic” in this document) multivariate α-stable densities that follow the superstatistical construction [19] (p. 78 Definition 2.5.1), the isotropic Cauchy is the only distribution that has a closed-form Probability Density Function (PDF). With a view towards developing a suitable working framework with tractable results, we tackle in this work the problem of testing the *n*-dimensional isotropic Cauchy distribution hypothesis (heavy-tailed dependent features) versus the *n*-dimensional isotropic Gaussian one (independent features). More specifically, given a sample of *n* observations sampled from a rotationally invariant density, we want to characterize the error limits of the LRT devices detecting whether the observations are circular Gaussian, with PDF denoted $p_{G}\left(\cdot \right)$, or circular Cauchy, with PDF $p_{C}\left(\cdot \right)$. We note that the proposed setup is substantially different from the standard one regarding two main aspects:

1. First, circular Cauchy vectors are statistically dependent (correlated) (whenever the usual notion of correlation is not well defined, such as for Cauchy variables, for example; we use the term “correlated” in this document to refer to statistical dependence) and not independent, as is the case for a Gaussian vector.
  This can be readily seen through multiplying two Cauchy PDFs, for example, and noting that the joint law is not an exclusive function of the norm. This indicates that a Cauchy vector with independent components is *not* circular.
2. Second, the relative entropy $D(p_{C}∥p_{G})$ is infinite, as shown in Appendix A.

Correspondingly, the results are found to deviate from the norm regarding two aspects:

- First, we show that the probability of error is not exponentially small with *n*. Namely, we prove that, under a Neyman–Pearson formulation and in the first order of the exponent, the leading term is linear for the error of the first kind ($P_{C|G}$) but only logarithmic for that of the second kind ($P_{G|C}$). A similar logarithmic behavior of the exponent of the leading term is shown for the Bayesian error. Whenever possible, we determine the multiplicative constants of the provided asymptotic expressions.
- Second, we observe that the two types of Bayesian errors do not possess the same asymptotic rate, which is at odds with the general assumption of a similar asymptotic behavior under IID statistics (see [1] (Th. 11.9.1]) for the discrete case). We postulate that this is due to the fact that the observations are correlated, and we support our observation by providing in Appendix B illustrative examples in which one of the two hypotheses is correlated.

The rest of this paper is organized as follows: we present the problem and the main results in the form of two theorems in Section 2. Derivation of the results use technical lemmas and are detailed in Section 3. Finally, Section 4 concludes the paper.

### Notations

We adopt the following conventions. For two asymptotically positive functions, $f\left(n\right)=o\left(g(n)\right)$ if for every c>0, f(n)≤cg(n) for *n* large enough. We also write f(n)≍g(n) if there exist $c_{1}>0$ and $c_{2}>0$ such that $f\left(n\right)\leq c_{1}g\left(n\right)$ and $f\left(n\right)\geq c_{2}g\left(n\right)$ for *n* large enough (this is sometimes written using the Knuth notation as $f\left(n\right)=\Theta \left(g(n)\right)$). Functions f(n) and g(n) are said to be “asymptotically equivalent” if $\lim_{n\to \infty }\frac{f(n)}{g(n)}=1$; we write f(n)∼g(n).

## 2. Problem Formulation and Main Results

We consider the binary hypothesis testing problem where, given a (large) number n>0 of observations, we need to decide between the two hypotheses:

$$\begin{array}{ccc} & & \left\{\begin{array}{cc} H_{G}: & Y^{n}\;\;\mathrm{circular}\;\;N\left(0,\sigma^{2}\right),\\ H_{C}: & Y^{n}\;\;\mathrm{circular}\;\;C\left(0,\gamma \right), \end{array}\right. \end{array}$$

where $Y^{n}$ circular $N(0,\sigma^{2})$ denotes a zero-mean multivariate isotropic Gaussian vector with covariance matrix $\Sigma =\sigma^{2}I_{n}$, $I_{n}$ being the n×n identity matrix. In contrast, $Y^{n}$ circular C(0,γ) represents an isotropic Cauchy vector with parameter γ, which can be written under the form $Y^{n}=\sqrt{A}\;G$, where $G={\left(G_{1},\cdots ,G_{n}\right)}^{t}$ is a zero-mean Gaussian vector with IID components each with variance $2\gamma^{2}$, and *A* is Lévy-distributed with a zero location parameter and a scale parameter equal to $\frac{1}{2}$ [19] (p. 78 Definition 2.5.1). We note that the components of the isotropic Cauchy vector are dependent.

Whether under a Bayesian risk formulation or a Neyman–Pearson criterion, the optimal device is known to be an LRT:

(1) $$\frac{p_{Y|H}\left(y|H_{G}\right)}{p_{Y|H}\left(y|H_{C}\right)}=\frac{p_{G}\left(y\right)}{p_{C}\left(y\right)}\begin{array}{c} H_{G}\\ \geq \\ <\\ H_{C} \end{array}\eta ,$$

for some appropriately chosen η>0. We note that as η increases, both $P_{C|G}$ and $\left(1-P_{G|C}\right)$ increase.

Our objective is twofold. First, we study the optimal Bayesian device and characterize its overall probability of error at large values of *n* via computing the asymptotic behavior of the probabilities of error of type I and II, as captured by $P_{C|G}$ and $P_{G|C}$, respectively. More specifically, if $\pi_{G}$ and $\pi_{C}=1-\pi_{G}$ denote the prior probabilities of $H_{G}$ and $H_{C}$, respectively, in Theorem 1, we show that as n→∞, the probability of error of the optimal device is

$$P_{e}=\pi_{G}P_{C|G}+\pi_{C}P_{G|C}≍\sqrt{\frac{\ln n}{n}}.$$

Second, we adopt the Neyman–Pearson criterion and answer the following question: let $P_{C|G}=\varepsilon$, for some ε∈(0,1). What is the asymptotic behavior of $P_{G|C}$ of the optimal device as n→∞? We show in Theorem 2 that

$$P_{G|C}≍\frac{1}{\sqrt{n}}.$$

We also answer the same question for the inverted problem: if $P_{G|C}=\varepsilon$, we show in Theorem 2 that

$$P_{C|G}=\frac{e^{-a(n)}}{\sqrt{n}},\;\mathrm{for}\;\mathrm{some}\;\;a\left(n\right)≍n.$$

For mathematical convenience and without loss of generality, we scale our observation vector $y^{n}$ by $\frac{1}{\gamma }$ and we denote *the constant* $\sqrt{2}\frac{\sigma }{\gamma }$ by $\xi =\sqrt{2}\frac{\sigma }{\gamma }$. The resulting equivalent problem is:

$$\begin{array}{ccc} & & \left\{\begin{array}{cc} H_{G}: & Y^{n}\;\;\mathrm{circular}\;\;N\left(0,\frac{\xi^{2}}{2}\right)\\ H_{C}: & Y^{n}\;\;\mathrm{circular}\;\;C\left(0,1\right). \end{array}\right. \end{array}$$

The PDFs $p_{Y|H}\left(y|H_{G}\right)$ and $p_{Y|H}\left(y|H_{C}\right)$ are given by

(2) $$\begin{array}{cc} p_{Y|H}\left(y|H_{G}\right) & =p_{G}\left(y\right)=\frac{1}{\pi^{\frac{n}{2}}\xi^{n}}e^{-\frac{r^{2}}{\xi^{2}}},\\ p_{Y|H}\left(y|H_{C}\right)\; & =\;p_{C}\left(y\right)\;=\;\frac{\Gamma \;\left(\frac{n+1}{2}\right)}{\pi^{\frac{n+1}{2}}}\frac{1}{{\left(1+r^{2}\right)}^{\frac{n+1}{2}}} \end{array}$$

where $r^{2}={∥y∥}^{2}$.

We state our main results in the form of Theorems 1 and 2. We also provide in Appendix D precise numerical computations that align with the results of Theorems 1 and 2.

In a Bayesian setting, the probability of error is determined in the following theorem:

**Theorem 1.**  *Consider a sequence of n observations and a binary decision problem with two hypotheses for their distribution: either circular (IID) Gaussian $N(0,\sigma^{2})$ with prior probability $0<\pi_{G}<1$ or circular (correlated) Cauchy C(0,γ) with prior probability $\pi_{C}=1-\pi_{G}$.*

*Let $\xi \;\hat{=}\;\sqrt{2}\frac{\sigma }{\gamma }$, $\tilde{\eta }\;\hat{=}\;\frac{\pi_{C}}{\pi_{G}}$ and $C\;\hat{=}\;\sqrt{\frac{1}{\tilde{\eta }}\;\frac{\xi }{2}}\;e^{\frac{1}{2\xi^{2}}}$. For the Maximum A Posteriori (MAP) detector, optimal under a Bayesian formulation,*

(3) $$\begin{array}{cc} \; & P_{G|C}∼\sqrt{\frac{2}{\pi }}\;\frac{1}{\tilde{\eta }}\sqrt{\frac{\ln\left(Cn\right)}{\left(Cn\right)}},\;\;P_{C|G}∼\sqrt{\frac{2}{\pi }}\;\frac{1}{\sqrt{\left(Cn\right)\ln\left(Cn\right)}}\\ \; & P_{e}=\pi_{G}P_{C|G}+\pi_{C}P_{G|C}∼\sqrt{\frac{2}{\pi }}\;\frac{\pi_{C}}{\tilde{\eta }}\sqrt{\frac{\ln\left(Cn\right)}{\left(Cn\right)}}. \end{array}$$

Note that the optimal error probabilities $P_{C|G}$ and $P_{G|C}$, the ones that minimize the Bayesian probability of error $P_{e}$, exhibit asymptotic behaviors that are *not symmetric*. That symmetry is a known property of the optimal Bayesian detector under IID observations: In a discrete binary hypothesis testing setup, whenever $X_{1},X_{2},\cdots ,X_{n}$ are IID according to *p* with prior probability π or *q* with prior probability (1−π), then both the type I and II error probabilities under the optimal test are asymptotically of the form $2^{-nC(p,q)}$, where C(p,q) is the Chernoff information [1]. This is not true in general under correlated settings as in Theorem 1. We provide additional examples in Appendix B, which also show that there are no guarantees under the correlated setup for $P_{e}$ to become infinitesimally small as the number of observations *n* increases.

**Theorem 2.**  *Consider a sequence of n observations and a binary decision problem with two hypotheses for their distribution: either circular (IID) Gaussian $N(0,\sigma^{2})$ or circular (correlated) Cauchy C(0,γ). Let $\xi \;\hat{=}\;\sqrt{2}\frac{\sigma }{\gamma }$, and consider an 0<ε<1.*

*Let Q(x) be the standard Gaussian Q-function (the Q(x) function is defined as $Q\left(x\right)=\frac{1}{\sqrt{2\pi }}\int_{x}^{\infty }e^{-\frac{u^{2}}{2}}\;du$) and $Q^{-1}\left(\cdot \right)$ be its inverse function. Under a Neyman–Pearson criterion, the optimal LRT detector is such that*

- *Case 1: whenever $P_{C|G}=\varepsilon$, $P_{G|C}∼\kappa_{0}\left(\epsilon \right)\frac{1}{\sqrt{n}}$, where $\kappa_{0}\left(\epsilon \right)\;\hat{=}\;\sqrt{\frac{2}{\pi }}\frac{2}{\xi }e^{-\frac{1}{\xi^{2}}}Q^{-1}\left(\frac{\epsilon }{2}\right)$.*
- *Case 2: whenever $P_{G|C}=\varepsilon$,*
$$\frac{\kappa_{1}\left(\epsilon \right)e^{-\kappa_{2}\left(\epsilon \right)n}}{\sqrt{n}}\leq P_{C|G}\leq \frac{\kappa_{3}\left(\epsilon \right)e^{-\kappa_{4}\left(\epsilon \right)n}}{\sqrt{n}},$$
  *for some $\kappa_{1}\left(\epsilon \right),\kappa_{2}\left(\epsilon \right),\kappa_{3}\left(\epsilon \right),\kappa_{4}\left(\epsilon \right)>0$.*

The results of Theorem 2 also depart from the standard results of the Chernoff–Stein lemma [1] (Thm. 11.8.3). That lemma states that under IID observations, setting the type I error probability to be no worse than ϵ>0, the type II error probability is asymptotically of the form $2^{-nD(p||q)}$, and hence exponentially decaying with *n* with a constant rate equal to the KL divergence between *p* and *q*, the two alternatives of the testing problem. In Theorem 2, Case 1 shows a polynomial decay and not an exponential one. We believe that this is due to having a correlated hypothesis. We prove in Appendix A that $D(P_{G}\left|\right|P_{C})∼\frac{1}{2}\ln n$, thus suggesting an asymptotic expression of the type II error probability of the form $2^{-D(p_{n}||q_{n})}$, where $p_{n}$, $q_{n}$ represent the joint probability distributions under *n* observations. This provides a generalization of the result of the Chernoff–Stein lemma under IID statistics.

The remainder of this document is dedicated to deriving the main results stated in Theorems 1 and 2.

## 3. Technical Proofs

### 3.1. Preliminaries

Define the left-hand side of Equation (1) as ℓ(r):

$$\ell \left(r\right)=\frac{p_{Y|H}\left(y|H_{G}\right)}{p_{Y|H}\left(y|H_{C}\right)}=\frac{\sqrt{\pi }}{\Gamma \;\left(\frac{n+1}{2}\right)\xi^{n}}e^{-\frac{r^{2}}{\xi^{2}}}{\left(1+r^{2}\right)}^{\frac{n+1}{2}}.$$

Whenever ℓ(r) is greater or equal to η, the LRT Equation (1) decides on the Gaussian hypothesis. As such, when computing $P_{C|G}$ and $\left(1-P_{G|C}\right)$, the larger the value of η is, the larger these probabilities.

**Figure 1:**
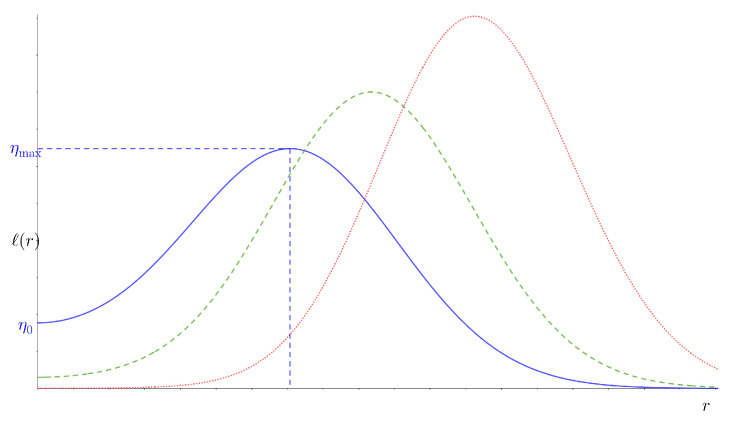
ℓ(r) for $\xi^{2}=1$, with n=5 (solid blue), n=8 (dashed green), and n=13 (pointed red).

Characterizing the LRT problem (1) is naturally governed by the variations of ℓ(r) and by the solution(s) of ℓ(r)=η. Given a value of ξ, ℓ(r) has a peak and takes the general form depicted in Figure 1 whenever $n>(2/\xi^{2}-1)$. Since we are interested in the asymptotic regime where *n* is very large, we assume that *n* is large enough for this condition to be true for any given value of ξ. Under this regime, ℓ(r) satisfies the following properties illustrated in Figure 1:

1. Asymptotically, $\ell \left(r\right)\to 0^{+}$ as r→∞.
2. Its *y*-intercept, which we denote by $\eta_{0}$, is equal to
  (4) $$\eta_{0}=\frac{\sqrt{\pi }}{\Gamma \;\left(\frac{n+1}{2}\right)\;\xi^{n}}.$$
3. It peaks at $r_{\max}$, reaching a value of $\eta_{\max}$:
  $$r_{\max}=\sqrt{\xi^{2}\frac{\left(n+1\right)}{2}-1}\;\&\;\eta_{\max}=e^{\frac{1}{\xi^{2}}}{\left(\frac{n+1}{2}\;\xi^{2}e^{-1}\right)}^{\frac{n+1}{2}}\eta_{0}.$$

Using Stirling’s approximation, one can show:

(5) $$\eta_{0}∼\frac{{\left(2e\right)}^{\frac{n-1}{2}}}{\xi^{n}{\left(n-1\right)}^{\frac{n}{2}}}\;\&\;\eta_{\max}∼e^{\frac{1}{\xi^{2}}}\frac{\xi }{2}\;\sqrt{n},$$

and since ξ is constant,

(6) $$\eta_{0}^{\frac{2}{n+1}}∼\frac{\left(2e\right)}{\xi^{2}}\;\frac{1}{n}\;\&\;\eta_{\max}^{\frac{2}{n+1}}∼1.$$

### 3.2. Operating Regime (OR)

Denote by

(7) $$a_{W}\left(n\right)\;\hat{=}\;-e^{-1}\;{\left(\frac{\eta }{\eta_{\max}}\right)}^{\frac{2}{n+1}}∼-e^{-1}\eta^{\frac{2}{n+1}},$$

where the approximation is due to Equation (6), and let $W_{0}\left(\cdot \right)$ and $W_{-1}\left(\cdot \right)$ denote the Lambert W function of branches 0 and −1, respectively. As can be seen in Figure 1, there are three regimes based on the value of the threshold η in Equation (1): $0<\eta \leq \eta_{0}$, $\eta_{0}<\eta <\eta_{\max}$, and $\eta \geq \eta_{\max}$. As it will turn out to be the case, the only “Operating Regime” (OR) for the problem at hand is the second one: $\eta_{0}<\eta <\eta_{\max}$, for which ℓ(r)=η has two solutions,

(8) $$\begin{array}{cc} r_{1}^{2} & =-\xi^{2}\left(\frac{n+1}{2}\right)W_{0}\left(a_{W}\left(n\right)\right)-1 \end{array}$$

(9) $$\begin{array}{cc} r_{2}^{2} & =-\xi^{2}\left(\frac{n+1}{2}\right)W_{-1}\left(a_{W}\left(n\right)\right)-1. \end{array}$$

We next compute $\left(1-P_{G|C}\right)$ and $P_{C|G}$ under the OR. We first compute $P_{G|C}$:

$$\begin{array}{cc} P_{G|C} & =\mathrm{Pr}\left(\hat{H}=H_{G}|H_{C}\right)=\int_{r_{1}}^{r_{2}}p_{Y|H}\left(y|H_{C}\right)\;dy \end{array}$$

(10) $$\begin{array}{cc} \; & \;\;\;=\frac{2}{\sqrt{\pi }}\frac{\Gamma \;\left(\frac{n+1}{2}\right)}{\Gamma \;\left(\frac{n}{2}\right)}\int_{r_{1}}^{r_{2}}{\left(1+r^{2}\right)}^{-\frac{n+1}{2}}r^{n-1}\;dr\\ \; & \;\;\;=\frac{2}{\sqrt{\pi }}\frac{\Gamma \;\left(\frac{n+1}{2}\right)}{\Gamma \;\left(\frac{n}{2}\right)}\int_{r_{1}}^{r_{2}}{\left(\frac{1}{r^{2}}+1\right)}^{-\frac{n+1}{2}}r^{-2}\;dr \end{array}$$

(11) $$\begin{array}{cc} \; & \;\;\;\;=\frac{1}{\sqrt{\pi }}\frac{\Gamma \;\left(\frac{n+1}{2}\right)}{\Gamma \;\left(\frac{n}{2}\right)}\int_{\frac{1}{r_{2}^{2}}}^{\frac{1}{r_{1}^{2}}}{\left(1+u\right)}^{-\frac{n+1}{2}}u^{-\frac{1}{2}}\;du, \end{array}$$

where we used the change of variable $u=\frac{1}{r^{2}}$ to write the last equality. As for $P_{C|G}$, we have:

(12) $$\begin{array}{cc} 1-P_{C|G} & =\mathrm{Pr}\left(\hat{H}=H_{G}|H_{G}\right)=\int_{r_{1}}^{r_{2}}p_{Y|H}\left(y|H_{G}\right)\;dy\\ \; & =\frac{2}{\xi^{n}\Gamma \;\left(\frac{n}{2}\right)}\int_{r_{1}}^{r_{2}}r^{n-1}e^{-\frac{r^{2}}{\xi^{2}}}\;dr=\frac{1}{\Gamma \;\left(\frac{n}{2}\right)}\int_{\frac{r_{1}^{2}}{\zeta^{2}}}^{\frac{r_{2}^{2}}{\zeta^{2}}}r^{\frac{n}{2}-1}e^{-r}\;dr\\ \; & =\frac{{\left(\frac{n}{2}-1\right)}^{\frac{n}{2}}}{\Gamma \;\left(\frac{n}{2}\right)}\int_{\frac{2r_{1}^{2}}{\zeta^{2}\left(n-2\right)}}^{\frac{2r_{2}^{2}}{\zeta^{2}\left(n-2\right)}}e^{\left(\frac{n}{2}-1\right)\left(\ln x-x\right)}\;dx, \end{array}$$

where Equation (12) is due to the change of variable $r=(\frac{n}{2}-1)x$.

In order to determine the asymptotic behavior of both $\left(1-P_{G|C}\right)$ and $P_{C|G}$ at large values of *n*, it is imperative to first analyze the growth rate of the intersection points under the OR. We identify three cases:

(i) $\eta^{\frac{2}{n+1}}\underset{n\to \infty }{⟶}1$: This includes, for example, the case of η fixed. Equation (7) implies that $a_{W}\left(n\right)\underset{n\to \infty }{⟶}-e^{-1}$ and [20] (pp. 350–351)
  $$W_{0}\left(z\right)∼-1+\sqrt{2\;(e\;z+1)},\;\;W_{-1}\left(z\right)∼-1-\sqrt{2\;(e\;z+1)},$$
  which gives
  (13) $$\begin{array}{c} \frac{r_{1}^{2}}{\xi^{2}}∼\frac{n+1}{2}\left(1-\delta_{n}\right)\;\&\;\frac{r_{2}^{2}}{\xi^{2}}∼\frac{n+1}{2}\left(1+\delta_{n}\right), \end{array}$$
  where
  (14) $$\begin{array}{cc} \delta_{n}\;\hat{=}\;\; & \sqrt{2}\;\sqrt{1-{\left(\frac{\eta }{\eta_{\max}}\right)}^{\frac{2}{n+1}}}∼2\;\sqrt{\frac{\ln\left(\frac{\eta_{\max}}{\eta }\right)}{n}}. \end{array}$$
  Note that in this case, $\delta_{n}\underset{n\to \infty }{⟶}0$ since $\lim_{n\to \infty }{\left(\frac{\eta }{\eta_{\max}}\right)}^{\frac{2}{n+1}}=1$.
(ii) $\eta^{\frac{2}{n+1}}\underset{n\to \infty }{⟶}\kappa$, 0<κ<1: In this case, Equation (7) implies that $a_{W}\left(n\right)\to \left(-\kappa \;e^{-1}\right)$ and
  (15) $$\begin{array}{cc} r_{1}^{2} & ∼-\xi^{2}\left(\frac{n+1}{2}\right)W_{0}\left(-\kappa \;e^{-1}\right)-1 \end{array}$$
  (16) $$\begin{array}{cc} r_{2}^{2} & ∼-\xi^{2}\left(\frac{n+1}{2}\right)W_{-1}\left(-\kappa \;e^{-1}\right)-1. \end{array}$$
(iii) $\eta^{\frac{2}{n+1}}\underset{n\to \infty }{⟶}0$, which implies $a_{W}\left(n\right)\underset{n\to \infty }{⟶}0^{-}$. Whenever $a_{W}\left(n\right)\to 0^{-}$, the following approximations hold [20] (p. 350):
  $$W_{0}\left[a_{W}\left(n\right)\right]∼a_{W}\left(n\right),\;\;W_{-1}\left[a_{W}\left(n\right)\right]∼\ln\left(-a_{W}\left(n\right)\right),$$
  Thus, Equations (8) and (9) give
  (17) $$\begin{array}{ccc} r_{1}^{2} & ∼ & \frac{\xi^{2}e^{-1}}{2}\;\left(n+1\right)\;\eta^{\frac{2}{n+1}}-1 \end{array}$$
  (18) $$\begin{array}{ccc} r_{2}^{2} & ∼ & \frac{\xi^{2}}{2}\;\left(n+1\right)\;\ln\eta^{-\frac{2}{n+1}}. \end{array}$$

For the remainder of this paper, we refer to the preceding three cases of the OR by (R-i), (R-ii), and (R-iii).

### 3.3. The Bayesian Detection Problem: Proofs

We start first by stating a technical lemma.

**Lemma 1.**  *Whenever ${\left(\frac{\eta }{\eta_{\max}}\right)}^{\frac{2}{n+1}}\underset{n\to \infty }{⟶}1$, the following hold:*

$$P_{G|C}∼\frac{2}{\sqrt{\pi }}\frac{\sqrt{\ln\left(\frac{\eta_{\max}}{\eta }\right)}}{\eta_{\max}},\;\;P_{C|G}∼2\;Q\left(\sqrt{2\ln\left(\frac{\eta_{\max}}{\eta }\right)}\right),$$

*where Q(·) is the standard Gaussian Q-function.*

**Proof.**  The detector is operating under Regime (R-i). Using the expression of $P_{G|C}$ in Equation (11), it is shown in Equation (A25) in Appendix C that:

$$\begin{array}{c} P_{G|C}∼\frac{1}{\sqrt{\pi }}\frac{\Gamma \;\left(\frac{n+1}{2}\right)}{\Gamma \;\left(\frac{n}{2}\right)}\frac{\sqrt{2}}{\sqrt{n+1}}\left[\Gamma \;\left(\frac{1}{2},\frac{n+1}{2r_{2}^{2}}\right)-\Gamma \;\left(\frac{1}{2},\frac{n+1}{2r_{1}^{2}}\right)\right], \end{array}$$

which gives

(19) $$\begin{array}{cc} P_{G|C}\; & ∼\sqrt{\frac{2}{\pi }}\frac{\Gamma \;\left(\frac{n+1}{2}\right)}{\Gamma \;\left(\frac{n}{2}\right)}\frac{1}{\sqrt{n+1}}\left[\frac{n+1}{2r_{2}^{2}}-\frac{n+1}{2r_{1}^{2}}\right]\Gamma^{\prime }\;\;\left(\frac{1}{2},\frac{1}{\xi^{2}}\right) \end{array}$$

(20) $$\begin{array}{cc} & \;∼\sqrt{\frac{2}{\pi }}\frac{\Gamma \;\left(\frac{n+1}{2}\right)}{\Gamma \;\left(\frac{n}{2}\right)}\frac{1}{\xi^{2}\sqrt{n+1}}\;\frac{2\delta_{n}}{1-\delta_{n}^{2}}\;\xi e^{-\frac{1}{\xi^{2}}} \end{array}$$

(21) $$\begin{array}{cc} & \;∼\frac{4}{\sqrt{\pi }}\frac{1}{\xi }e^{-\frac{1}{\xi^{2}}}\sqrt{\frac{\ln\left(\frac{\eta_{\max}}{\eta }\right)}{n}} \end{array}$$

(22) $$\begin{array}{cc} & \;∼\frac{2}{\sqrt{\pi }}\frac{\sqrt{\ln\left(\frac{\eta_{\max}}{\eta }\right)}}{\eta_{\max}}, \end{array}$$

where we used Equation (5) in order to write Equation (22). Equation (19) is due to Taylor’s approximation of the upper incomplete Gamma function $\Gamma \;\left(\frac{1}{2},x\right)$ around $x=\frac{1}{\xi^{2}}$, and we used the fact that $\frac{d}{dx}\Gamma \left(m,x\right)=-x^{m-1}e^{-x}$ to write Equation (20). Equation (21) is due to Equation (14) and to the fact that

$$\frac{\Gamma \;\left(\frac{n+1}{2}\right)}{\Gamma \;\left(\frac{n}{2}\right)}∼\sqrt{\frac{n}{2}}.$$

This concludes the first part of the proof. When it comes to $\left(1-P_{C|G}\right)$, its expression is given by Equation (12):

(23) $$\begin{array}{cc} \; & 1-P_{C|G}=\frac{{\left(\frac{n}{2}-1\right)}^{\frac{n}{2}}}{\Gamma \;\left(\frac{n}{2}\right)}\int_{\frac{2r_{1}^{2}}{\xi^{2}\left(n-2\right)}}^{\frac{2r_{2}^{2}}{\xi^{2}\left(n-2\right)}}e^{\left(\frac{n}{2}-1\right)\left(\ln x-x\right)}\;dx\\ \; & ∼\frac{{\left(\frac{n}{2}-1\right)}^{\frac{n}{2}}e^{-(\frac{n}{2}-1)}}{\Gamma \;\left(\frac{n}{2}\right)}\int_{\frac{2r_{1}^{2}}{\xi^{2}\left(n-2\right)}}^{\frac{2r_{2}^{2}}{\xi^{2}\left(n-2\right)}}e^{-\left(\frac{n}{2}-1\right)\frac{{\left(x-1\right)}^{2}}{2}}\;dx\\ \; & =\frac{{\left(\frac{n}{2}-1\right)}^{\frac{n}{2}}e^{-(\frac{n}{2}-1)}}{\Gamma \;\left(\frac{n}{2}\right)}\sqrt{\frac{4\pi }{n-2}}\left[Q\;\left(\frac{\frac{2r_{1}^{2}}{\xi^{2}\left(n-2\right)}-1}{\sqrt{\frac{2}{n-2}}}\right)-Q\left(\frac{\frac{2r_{2}^{2}}{\xi^{2}\left(n-2\right)}-1}{\sqrt{\frac{2}{n-2}}}\right)\right] \end{array}$$

(24) $$\begin{array}{cc} & \;∼1-2Q\left(\sqrt{2\ln\left(\frac{\eta_{\max}}{\eta }\right)}\right), \end{array}$$

where, in order to write the last equation, we used Stirling’s approximation

$$\Gamma \;\left(\frac{n}{2}\right)∼\sqrt{2\pi \left(\frac{n}{2}-1\right)}{\left(\frac{\frac{n}{2}-1}{e}\right)}^{\frac{n}{2}-1},$$

and the fact that:

$$\frac{\frac{2r_{1}^{2}}{\xi^{2}\left(n-2\right)}-1}{\sqrt{\frac{2}{n-2}}}∼-\sqrt{2\ln\left(\frac{\eta_{\max}}{\eta }\right)},\;\;\frac{\frac{2r_{2}^{2}}{\xi^{2}\left(n-2\right)}-1}{\sqrt{\frac{2}{n-2}}}∼\sqrt{2\ln\left(\frac{\eta_{\max}}{\eta }\right)},$$

as given by Equations (13) and (14). Equation (23) is due to applying the Laplace approximation method [21] around x=1. This concludes the proof of the lemma. □

Proof of Theorem 1 (Stated in Section 2)

**Proof.**  Since the MAP detector is an LRT Equation (1) for $\tilde{\eta }=\frac{\pi_{C}}{\pi_{G}}>0$, for *n* large enough and $\eta =\tilde{\eta }$ fixed, the detector Equation (1) will be operating under Regime (R-i), and hence, the expressions of $P_{G|C}$ and $P_{C|G}$ of Lemma 1 do apply. Replacing $\eta_{\max}$ by its asymptotic behavior as characterized by Equation (5) yields the required expression for $P_{G|C}$. The same applies to $P_{C|G}$ after using the following approximation [22]:

(25) $$Q\left(x\right)\underset{x\to \infty }{∼}\frac{1}{\sqrt{2\pi }}\frac{e^{-\frac{x^{2}}{2}}}{x}.$$

This concludes the proof of Theorem 1. □

### 3.4. The Neyman–Pearson Formulation: Proofs

When it comes to the Neyman–Pearson criteria, we first estalish the following lemma:

**Lemma 2.**  *For n large enough, the optimal Neyman–Pearson LRT detectors operate:*

- *under Regime (R-i)* *whenever* $P_{C|G}=\epsilon$*,*
- *under Regime (R-ii) whenever* $P_{G|C}=\epsilon$.

**Proof.**  Since $P_{C|G}$ increases with η, in order for $P_{C|G}$ to be equal to ε∈(0,1) as *n* grows to infinity, then η must increase “as fast as $\eta_{\max}$” with *n*, for otherwise, $P_{C|G}$ would go to zero, as implied by our analysis in Lemma 1 and our observations in Section 3. Moreover, since $\eta_{\max}^{\frac{2}{n+1}}\underset{n\to \infty }{⟶}1$ Equation (5), and $\eta \leq \eta_{\max}$, then by inspection, one can see that necessarily $\eta^{\frac{2}{n+1}}\underset{n\to \infty }{⟶}1$, which corresponds to Regime (R-i).

Similarly, $P_{G|C}$ is decreasing with η and for a fixed η$P_{G|C}\underset{n\to \infty }{⟶}0$. In order for $P_{G|C}$ to be equal to ε, η must decrease with *n*. We prove next that Regime (R-iii) (and a fortiori $\eta <\eta_{0}$) is not possible, for otherwise, $P_{G|C}\underset{n\to \infty }{⟶}1$. In fact, Equation (A24) in Appendix C gives a lowerbound on $P_{G|C}$:

$$P_{G|C}\geq \frac{1}{\sqrt{\pi }}\frac{\Gamma \;\left(\frac{n+1}{2}\right)}{\Gamma \;\left(\frac{n}{2}\right)}\sqrt{\frac{2}{n+1}}\;\left[\Gamma \;\left(\frac{1}{2},\frac{n+1}{2r_{2}^{2}}\right)-\Gamma \;\left(\frac{1}{2},\frac{n+1}{2r_{1}^{2}}\right)\right]\underset{n\to \infty }{⟶}\frac{1}{\sqrt{\pi }}\;\Gamma \;\left(\frac{1}{2},0\right)-0=1,$$

where the limit result is due to Stirling’s identity and to the asymptotic behavior of $r_{1}^{2}$ and $r_{2}^{2}$ under Regime (R-iii) according to Equations (17) and (18). Furthermore, we show that η must be under Regime (R-ii) by ruling out Regime (R-i) as well. Indeed, under case (R-i), the condition of Lemma 1 is satisfied, and hence,

$$P_{G|C}∼\frac{2}{\sqrt{\pi }}\frac{\sqrt{\ln\left(\frac{\eta_{\max}}{\eta }\right)}}{\eta_{\max}}\underset{n\to \infty }{⟶}0,$$

where we used the fact that $\eta_{\max}≍\sqrt{n}$ Equation (5). This rules out the Regime (R-i), thus proving our assertion. □

Proof of Theorem 2 (Stated in Section 2)

**Proof.**  Let 0<ϵ<1. We begin with the proof of Case 1. Whenever $P_{C|G}=\varepsilon$, the results of Lemma 2 imply that Regime (R-i) is the operating regime. Therefore, we use the result of Lemma 1 to write the expression of $P_{C|G}$:

(26) $$\lim_{n\to \infty }2\;Q\left(\sqrt{2\ln\left(\frac{\eta_{\max}}{\eta }\right)}\right)=\varepsilon \;\Rightarrow \;\lim_{n\to \infty }\ln\left(\frac{\eta_{\max}}{\eta }\right)=\frac{1}{2}{\left(Q^{-1}\left(\frac{\epsilon }{2}\right)\right)}^{2},$$

where $Q^{-1}\left(\cdot \right)$ is the inverse *Q*-function. Using the expression of $P_{G|C}$ as given in Lemma 1, we obtain:

(27) $$P_{G|C}∼\frac{2}{\sqrt{\pi }}\frac{\sqrt{\ln\left(\frac{\eta_{\max}}{\eta }\right)}}{\eta_{\max}}∼\sqrt{\frac{2}{\pi }}\frac{2}{\xi }e^{-\frac{1}{\xi^{2}}}Q^{-1}\left(\frac{\epsilon }{2}\right)\frac{1}{\sqrt{n}},$$

where the last relation follows from Equations (5) and (26). This completes the proof of Case 1.

We move now to proving Case 2 of the theorem. As shown in Lemma 2, Regime (R-ii) is the OR, and therefore, the expression given by Lemma 1 does not hold. We proceed by writing $P_{C|G}$ as given by Equation (12):

(28) $$\begin{array}{cc} & P_{C|G}=\frac{{\left(\frac{n}{2}-1\right)}^{\frac{n}{2}}}{\Gamma \;\left(\frac{n}{2}\right)}\left[\int_{0}^{\frac{2r_{1}^{2}}{\left(n-2\right)\xi^{2}}}\;e^{\left(\frac{n}{2}-1\right)\left(\ln x-x\right)}\;dx+\int_{\frac{2r_{2}^{2}}{\left(n-2\right)\xi^{2}}}^{\infty }e^{\left(\frac{n}{2}-1\right)\left(\ln x-x\right)}\;dx\right]\\ \; & \;\;\leq \frac{{\left(\frac{n}{2}-1\right)}^{\frac{n}{2}}e^{-\left(\frac{n}{2}-1\right)}}{\Gamma \;\left(\frac{n}{2}\right)}\left[\int_{0}^{\frac{2r_{1}^{2}}{\left(n-2\right)\xi^{2}}}e^{-\left(\frac{n}{2}-1\right)\frac{{\left(x-1\right)}^{2}}{2}}\;dx+\;\int_{\frac{2r_{2}^{2}}{\left(n-2\right)\xi^{2}}}^{\infty }e^{-\left(\frac{n}{2}-1\right)K\left(\epsilon \right)\left(x-1\right)}\;dx\right]\\ \; & \;\;=\frac{{\left(\frac{n}{2}-1\right)}^{\frac{n}{2}}e^{-\left(\frac{n}{2}-1\right)}}{\Gamma \;\left(\frac{n}{2}\right)}\left[\sqrt{\frac{2\pi }{\frac{n}{2}-1}}\left(Q\;\left(\frac{-1}{\sqrt{\frac{2}{n-2}}}\right)-Q\;\left(\frac{\frac{2r_{1}^{2}}{\left(n-2\right)\xi^{2}}-1}{\sqrt{\frac{2}{n-2}}}\right)\right)\right. \end{array}$$

$$\begin{array}{cc} \; & \left.\;\;\;\;\;\;\;\;\;\;\;\;\;+\frac{e^{-\left(\frac{n}{2}-1\right)K\left(\epsilon \right)\left(\frac{2r_{2}^{2}}{\left(n-2\right)\xi^{2}}-1\right)}}{K\left(\epsilon \right)\left(\frac{n}{2}-1\right)}\right] \end{array}$$

(29) $$\begin{array}{cc} \; & ∼\frac{1}{\sqrt{\pi }}\;\frac{1}{\sqrt{n-2}}\left[\;\frac{e^{-\frac{\left(n-2\right){\left(1+W_{0}\left(-e^{-1}\kappa \left(\epsilon \right)\right)\right)}^{2}}{4}}}{1+W_{0}\left(-e^{-1}\kappa \left(\epsilon \right)\right)}-e^{-\frac{n-2}{4}}+\frac{e^{-\left(\frac{n}{2}-1\right)K\left(\epsilon \right)\left[-1-W_{-1}\left(-e^{-1}\kappa \left(\epsilon \right)\right)\right]}}{K\left(\epsilon \right)\sqrt{\pi }\sqrt{n-2}}\right]\\ \; & ∼\frac{\kappa_{3}\left(\epsilon \right)e^{-\kappa_{4}\left(\epsilon \right)n}}{\sqrt{n}}, \end{array}$$

where $\kappa_{3}\left(\epsilon \right)>0$, $\kappa_{4}\left(\epsilon \right)>0$ can be a judiciously chosen function of the different parameters in Equation (29). Equation (29) is due to Stirling’s approximation, to the approximation in Equation (25), and to the fact that

$$\lim_{n\to \infty }\frac{2r_{1}^{2}}{\left(n-2\right)\xi^{2}}=-W_{0}\left(-e^{-1}\kappa \left(\epsilon \right)\right)<1,\;\&\;\lim_{n\to \infty }\frac{2r_{2}^{2}}{\left(n-2\right)\xi^{2}}=-W_{-1}\left(-e^{-1}\kappa \left(\epsilon \right)\right)>1,$$

given the asymptotic behavior of $r_{1}^{2}$ and $r_{2}^{2}$ under case (R-ii) as given by Equations (15) and (16), and where $\kappa \left(\epsilon \right)\;\hat{=}\;\lim_{n\to \infty }\eta^{\frac{2}{n+1}}$, 0<κ(ϵ)<1. Equation (28) is due to the following identities:

$$\ln x-x\leq -1-\frac{{\left(x-1\right)}^{2}}{2}\;0\leq x\leq 1,$$

and for every c>1, there exits a K∈(0,1) such that:

lnx−x≤−1−K(x−1)c≤x.

Finally, we provide a lowerbound on $P_{C|G}$:

(30) $$\begin{array}{cc} & \;\;\;\;\;\;\;\;\;P_{C|G}=\frac{1}{\Gamma \;\left(\frac{n}{2}\right)}\;\left[\int_{0}^{\frac{r_{1}^{2}}{\xi^{2}}}\;r^{\frac{n}{2}-1}e^{-r}\;dr+\int_{\frac{r_{2}^{2}}{\xi^{2}}}^{\infty }\;r^{\frac{n}{2}-1}e^{-r}\;dr\right]\geq \frac{1}{\Gamma \;\left(\frac{n}{2}\right)}\int_{\frac{r_{2}^{2}}{\xi^{2}}}^{\infty }r^{\frac{n}{2}-1}e^{-r}\;dr\\ \; & \;\;\;\;\;\;\;\;\;\;\;=\frac{{\left(\frac{n}{2}-1\right)}^{\frac{n}{2}}}{\Gamma \;\left(\frac{n}{2}\right)}\int_{\frac{2r_{2}^{2}}{\left(n-2\right)\xi^{2}}}^{\infty }e^{\left(\frac{n}{2}-1\right)\left(\ln x-x\right)}\;dx\\ \; & \;\;\;\;\;\;\;\;\;\;\;\geq \frac{{\left(\frac{n}{2}-1\right)}^{\frac{n}{2}}e^{-\left(\frac{n}{2}-1\right)}}{\Gamma \;\left(\frac{n}{2}\right)}\;\int_{\frac{2r_{2}^{2}}{\left(n-2\right)\xi^{2}}}^{\infty }\;e^{-\left(\frac{n}{2}-1\right)\frac{{\left(x-1\right)}^{2}}{2}}dx\\ \; & \;\;\;\;\;\;\;\;\;\;\;=\frac{{\left(\frac{n}{2}-1\right)}^{\frac{n}{2}}e^{-\left(\frac{n}{2}-1\right)}}{\Gamma \;\left(\frac{n}{2}\right)}\sqrt{\frac{2\pi }{\frac{n}{2}-1}}Q\;\left(\frac{\frac{2r_{2}^{2}}{\left(n-2\right)\xi^{2}}-1}{\sqrt{\frac{2}{n-2}}}\right) \end{array}$$

(31) $$\begin{array}{cc} \; & ∼\frac{1}{\sqrt{\pi }}\;\frac{1}{\sqrt{n-2}}\;\frac{e^{-\frac{\left(n-2\right){\left[-1-W_{-1}\left(-e^{-1}\kappa \left(\epsilon \right)\right)\right]}^{2}}{4}}}{-1-W_{-1}\left(-e^{-1}\kappa \left(\epsilon \right)\right)}\\ \; & =\frac{\kappa_{1}\left(\epsilon \right)e^{-\kappa_{2}\left(\epsilon \right)n}}{\sqrt{n}}, \end{array}$$

for some $\kappa_{1}\left(\epsilon \right),\kappa_{2}\left(\epsilon \right)>0$. Equation (30) is due to the fact that $\ln x-x\geq -1-\frac{{\left(x-1\right)}^{2}}{2}$ for x≥1, and Equation (31) is justified in a similar manner as done for Equation (29). □

## 4. Conclusions

It is known that, in a discrete setup and under IID observations, the optimal Bayesian error probability $P_{e}$ is attained with type I and II error probabilities exponentially decaying with the number of observations *n* and both having the same exponent equal to the Chernoff information. Moreover, that of the error of the second kind $P_{G|C}$ goes to zero when n→∞ according to $2^{-nD(p∥q)}$, where *p* and *q* are the probability laws under the two different hypotheses. In some cases, a similar observation can be inferred whenever *p* and *q* are continuous [2]. Though the results presented in this paper are for a specific problem on hypothesis testing between two isotropic probability laws, they are rather interesting on their own and are “non-standard” when compared to the IID “Gaussian”-centric case. Our findings suggest new insights on the hypothesis testing problem:

1. Under a Bayesian setup, the type I and II error probabilities that give the fastest decay of $P_{e}$ exhibit different asymptotic behaviors in general.
2. Under a Neyman–Pearson setup, in a generalization to the IID case, the leading term in the exponent might be possibly governed by the behavior of $D(p_{n}∥q_{n})$ as a function of *n* whenever the relative entropy is finite. This extension is currently being investigated by the authors. Note that, under the IID case, we have $D\left(p_{n}∥q_{n}\right)=nD\left(p∥q\right)$, which recovers the standard result of the IID setup.
3. In general, $D(p_{n}∥q_{n})$ is not linear in *n*. When $p_{n}$ is circular (IID) $N(0,\sigma^{2})$ and $q_{n}$ is circularC(0,γ), we show in Appendix A that $D\left(p_{n}∥q_{n}\right)≍\ln\sqrt{n}$, which corroborates the previous point as per the results of Theorem 2 for $P_{G|C}$.
4. Whenever $D(p_{n}∥q_{n})$ is not finite, the term in the exponent is not linear: it was found in this setup to be of the form $-[a\left(n\right)+\frac{1}{2}\ln n]$ for some a(n)≍n.

## Figures and Tables

**Figure A1:**
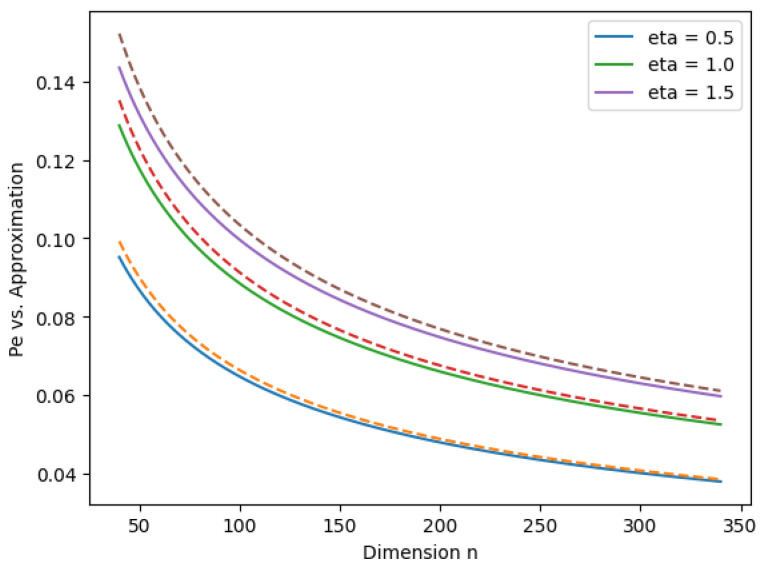
$P_{e}$ vs. *n* for η=0.5,1,1.5. Solid line: numerical computations. Dashed line: approximate expression.

**Figure A2:**
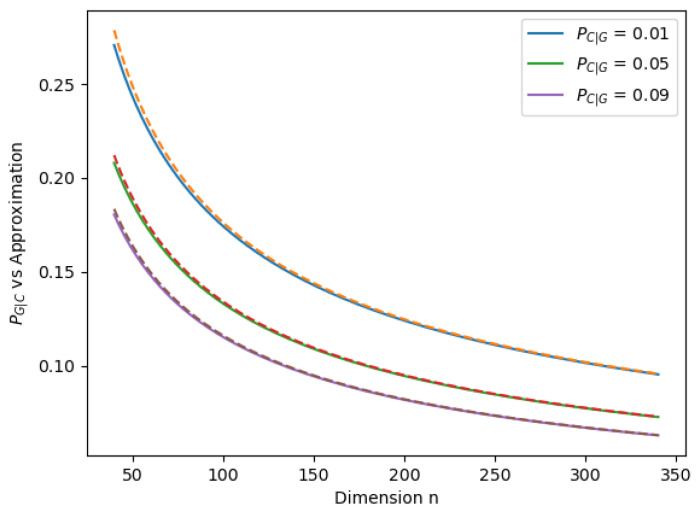
$P_{G|C}$ vs. n for $P_{C|G}=0.01,0.05,0.09$. Solid line: numerical computations. Dashed line: approximate expression.

**Figure A3:**
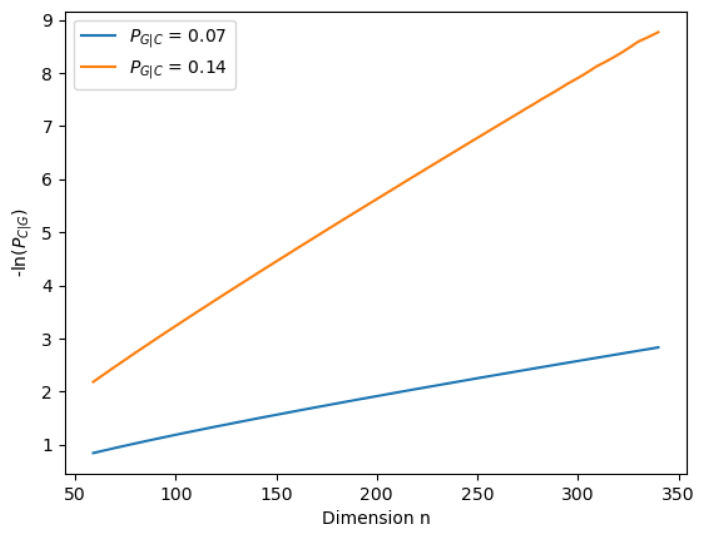
$-\ln P_{C|G}$ vs. n for $P_{G|C}=0.07,0.14$.

## Data Availability

No new data were created or analyzed in this study. Data sharing is not applicable to this article.

## Abbreviations

The following abbreviations are used in this manuscript:

LRT	Likelihood Ratio Tests
KL	Kullback–Leibler
IID	Independent and Identically Distributed
RV	Random Variable
PDF	Probability Density Function
MAP	Maximum A Posteriori
OR	Operating Regime
ML	Maximum Likelihood

## Appendix A. The KL Divergence

We characterize in this appendix the behavior at large values of *n* of the relative entropy between an IID *n*-dimensional zero-mean and variance $\frac{\xi^{2}}{2}$ Gaussian distribution $p_{G}\;\hat{=}\;N\left(0,\frac{\xi^{2}}{2}\right)$ and an isotropic *n*-dimensional Cauchy distribution $p_{C}$ with a scale parameter γ=1. Our main result shows that $D\left(p_{G}∥p_{C}\right)∼\frac{1}{2}\ln n$. However, before proceeding with the proof, we highlight the important fact that $D(p_{C}∥p_{G})$ is infinite. Since both $p_{G}\left(x\right)$ and $p_{C}\left(x\right)$ only depend on r=∥x∥, we abuse the notation in what follows and write $p_{G}\left(r\right)$ and $p_{C}\left(r\right)$ instead to highlight this dependency without renaming the functions.

$$\begin{array}{cc} D(p_{C}∥p_{G}) & =-h\left(p_{C}\right)-\int_{R^{n}}p_{C}\left(x\right)\ln p_{G}\left(x\right)\;dx=-h\left(p_{C}\right)+\ln\left(\pi^{\frac{n}{2}}\xi^{n}\right)+\frac{1}{\xi^{2}}\int_{R^{n}}r^{2}p_{C}\left(r\right)\;dx\\ \; & =-h\left(p_{C}\right)+\ln\left(\pi^{\frac{n}{2}}\xi^{n}\right)+\frac{1}{\xi^{2}}\frac{2\pi^{\frac{n}{2}}}{\Gamma \;\left(\frac{n}{2}\right)}\int_{0}^{\infty }r^{n+1}p_{C}\left(r\right)\;dr=\infty , \end{array}$$

where, in order to write the last equality, we used the fact that $h(p_{C})<\infty$ and that $p_{C}\left(r\right)≍r^{-n-1}$ as r→+∞, as given by Equation (2).

Back to computing $D(p_{G}∥p_{C})$, we assume n≥2 and we write:

(A1) $$\begin{array}{cc} D(p_{G}∥p_{C}) & =-h\left(p_{G}\right)-\int_{R^{n}}p_{G}\left(x\right)\ln p_{C}\left(x\right)\;dx\\ \; & =-\frac{n}{2}\ln\left(\pi e\xi^{2}\right)-\ln\frac{\Gamma \left(\frac{n+1}{2}\right)}{\pi^{\frac{n+1}{2}}}+\frac{n+1}{2\pi^{\frac{n}{2}}\xi^{n}}\int_{R^{n}}e^{-\frac{r^{2}}{\xi^{2}}}\ln\left(1+r^{2}\right)\;dx\\ \; & =-\frac{n}{2}\ln\left(\pi e\xi^{2}\right)-\ln\frac{\Gamma \left(\frac{n+1}{2}\right)}{\pi^{\frac{n+1}{2}}}+\frac{n+1}{\xi^{n}\Gamma \left(\frac{n}{2}\right)}\int_{0}^{\infty }e^{-\frac{r^{2}}{\xi^{2}}}\ln\left(1+r^{2}\right)r^{n-1}\;dr\\ \; & =-\frac{n}{2}\ln\left(\pi e\xi^{2}\right)-\ln\frac{\Gamma \left(\frac{n+1}{2}\right)}{\pi^{\frac{n+1}{2}}}+\frac{n+1}{2}\;I_{n}, \end{array}$$

where

(A2) $$\begin{array}{cc} I_{n} & =\frac{1}{\Gamma \left(\frac{n}{2}\right)}\int_{0}^{\infty }e^{-r}\ln\left(1+\xi^{2}r\right)r^{\frac{n}{2}-1}\;dr\\ \; & =\frac{{\left(\frac{n}{2}-1\right)}^{\frac{n}{2}}}{\Gamma \left(\frac{n}{2}\right)}\int_{0}^{\infty }e^{\left(\frac{n}{2}-1\right)\left(\ln r-r\right)}\ln\left[1+\left(\frac{n}{2}-1\right)\xi^{2}r\right]dr\\ \; & =I_{1n}+I_{2n}+I_{3n}, \end{array}$$

where $I_{1n}$, $I_{2n}$, $I_{3n}$ are a partition of $I_{n}$ on the respective disjoint sets: $B_{1}=\left(0,1-\epsilon_{n}\right)\cup \left(1+\epsilon_{n},R\right]$, $B_{2}=\left[1-\epsilon_{n},1+\epsilon_{n}\right]$, and $B_{3}=\left(R,\infty \right)$ for some R>0 and $\epsilon_{n}=\left(\frac{\ln n}{\sqrt{n}}\right)$. Note that $\epsilon_{n}\to 0$ as n→∞, and *R* is a constant to be chosen large enough as determined hereafter.

We first consider $I_{1n}$ and show that it is $o\left(\frac{\ln n}{n}\right)$. For that matter, it is enough to show that the integrand is $o\left(\frac{\ln n}{n}\right)$. Indeed, for $r\in B_{1}$, we have:

(A3) $$\begin{array}{cc} \frac{{\left(\frac{n}{2}-1\right)}^{\frac{n}{2}}}{\Gamma \left(\frac{n}{2}\right)}e^{\left(\frac{n}{2}-1\right)\left(\ln r-r\right)}\ln\left[1\;+\;\left(\frac{n}{2}-1\right)\xi^{2}r\right]\; & \leq \frac{{\left(\frac{n}{2}-1\right)}^{\frac{n}{2}}}{\Gamma \left(\frac{n}{2}\right)}e^{\left(\frac{n}{2}-1\right)\left[-1-\frac{\epsilon_{n}^{2}}{3}\right]}\ln\left[1\;+\;\left(\frac{n}{2}-1\right)\xi^{2}r\right] \end{array}$$

(A4) $$\begin{array}{cc} \; & \;\;\;\;\;\;\;\;\;\;\;\;\;\;\;∼\;\frac{{\left(\frac{n}{2}-1\right)}^{\frac{1}{2}}}{\sqrt{2\pi }}e^{\left(\frac{n}{2}-1\right)\left[-\frac{\epsilon_{n}^{2}}{3}\right]}\ln\left[\left(\frac{n}{2}-1\right)\xi^{2}r\right] \end{array}$$

(A5) $$\begin{array}{cc} \; & \;\;\;=\;o\left(\frac{\ln n}{n}\right), \end{array}$$

where we used the fact that $\ln r-r+1<\ln\left(1+\epsilon_{n}\right)-\epsilon_{n}<-\frac{\epsilon_{n}^{2}}{3}$ on $B_{1}$ to write Equation (A3) and Stirling’s approximation of the Gamma function to write Equation (A4).

Regarding $I_{3n}>0$, we have:

(A6) $$\begin{array}{cc} I_{3n} & =\frac{{\left(\frac{n}{2}-1\right)}^{\frac{n}{2}}}{\Gamma \left(\frac{n}{2}\right)}\int_{R}^{\infty }e^{\left(\frac{n}{2}-1\right)\left(\ln r-r\right)}\ln\left[1+\left(\frac{n}{2}-1\right)\xi^{2}r\right]\;dr\\ \; & \leq \frac{{\left(\frac{n}{2}-1\right)}^{\frac{n}{2}}}{\Gamma \left(\frac{n}{2}\right)}\int_{R}^{\infty }e^{-\left(\frac{n}{2}-1\right)\frac{r}{2}}\left[\ln\left[\left(\frac{n}{2}-1\right)\xi^{2}\right]+r\right]\;dr\\ \; & =\frac{{\left(\frac{n}{2}-1\right)}^{\frac{n}{2}}}{\Gamma \left(\frac{n}{2}\right)}\left[\ln\left[\left(\frac{n}{2}-1\right)\xi^{2}\right]\frac{2}{\frac{n}{2}-1}e^{-\left(\frac{n}{2}-1\right)\frac{R}{2}}+\frac{2}{\frac{n}{2}-1}R\;e^{-\left(\frac{n}{2}-1\right)\frac{R}{2}}\right. \end{array}$$

(A7) $$\begin{array}{cc} \; & \left.\;\;\;\;\;\;\;\;\;\;\;\;\;\;\;\;\;+{\left(\frac{2}{\frac{n}{2}-1}\right)}^{2}e^{-\left(\frac{n}{2}-1\right)\frac{R}{2}}\right] \end{array}$$

(A8) $$\begin{array}{cc} \; & \;\;\;\;∼\frac{{\left(\frac{n}{2}-1\right)}^{\frac{1}{2}}}{\sqrt{2\pi }}e^{\left(\frac{n}{2}-1\right)}e^{-\left(\frac{n}{2}-1\right)\frac{R}{2}}\left[\ln\left[\left(\frac{n}{2}-1\right)\xi^{2}\right]\frac{2}{\frac{n}{2}-1}+\frac{2}{\frac{n}{2}-1}R\;+{\left(\frac{2}{\frac{n}{2}-1}\right)}^{2}\right]\\ \; & \;\;\;\;=o\left(\frac{\ln n}{n}\right), \end{array}$$

which is true for R>2. Equation (A6) is justified by the fact that $1+\left(\frac{n}{2}-1\right)\xi^{2}r<\left(\frac{n}{2}-1\right)\xi^{2}e^{r}$ for n>2 and $r\in B_{3}$ with *R* large enough. Equation (A7) is due to integration by parts.

Finally, considering $I_{2n}$, we obtain:

(A9) $$\begin{array}{cc} & \;I_{2n}=\frac{{\left(\frac{n}{2}-1\right)}^{\frac{n}{2}}}{\Gamma \left(\frac{n}{2}\right)}\int_{1-\epsilon_{n}}^{1+\epsilon_{n}}\;e^{\left(\frac{n}{2}-1\right)\left(\ln r-r\right)}\ln\left[1+\left(\frac{n}{2}-1\right)\xi^{2}r\right]\;dr\\ \; & \;\geq \left[\frac{{\left(\frac{n}{2}-1\right)}^{\frac{n}{2}}}{\Gamma \left(\frac{n}{2}\right)}\int_{1-\epsilon_{n}}^{1+\epsilon_{n}}e^{\left(\frac{n}{2}-1\right)\left(\ln r-r\right)}\;dr\right]\ln\left[1+\left(\frac{n}{2}-1\right)\xi^{2}\left(1-\epsilon_{n}\right)\right] \end{array}$$

(A10) $$\begin{array}{cc} \; & \;\;\geq \;\left[\frac{{\left(\frac{n}{2}-1\right)}^{\frac{n}{2}}e^{-\left(\frac{n}{2}-1\right)}}{\Gamma \left(\frac{n}{2}\right)}\;\int_{1-\epsilon_{n}}^{1+\epsilon_{n}}\;e^{-\left(\frac{n}{2}-1\right)\left[\frac{1}{2}{\left(r-1\right)}^{2}+\frac{{2|r-1|}^{3}}{3}\right]}dr\right]\ln\left[1+\left(\frac{n}{2}-1\right)\xi^{2}\left(1-\epsilon_{n}\right)\right]\\ \; & \;\;\geq \frac{{\left(\frac{n}{2}-1\right)}^{\frac{n}{2}}e^{-\left(\frac{n}{2}-1\right)}}{\Gamma \left(\frac{n}{2}\right)}\sqrt{\frac{2\pi }{\frac{n}{2}-1}}e^{-\left(\frac{n}{2}-1\right)\frac{2\epsilon_{n}^{3}}{3}}\left[1-2Q\left(\epsilon_{n}\sqrt{\frac{n}{2}-1}\right)\right] \end{array}$$

(A11) $$\begin{array}{cc} \; & \;\;\;\;\;\;\;\;\;\;\times \ln\left[1+\left(\frac{n}{2}-1\right)\xi^{2}\left(1-\epsilon_{n}\right)\right] \end{array}$$

(A12) $$\begin{array}{cc} \; & \;∼\ln\left(\frac{n}{2}\;\xi^{2}\;\right), \end{array}$$

where, again, we used Stirling’s approximation of the Gamma function in order to write Equation (A12). Equation (A10) is due to the fact that $\ln\left(r\right)-r+1\geq -{\left(r-1\right)}^{2}/2-2{\left|r-1\right|}^{3}/3$ around r=1, and Equation (A11) is justified by the fact that the term ${\left|r-1\right|}^{3}\leq \epsilon_{n}^{3}$ whenever $\left|r-1\right|\leq \epsilon_{n}$. A similar upperbound can be readily obtained by replacing $\ln\left[1+\left(\frac{n}{2}-1\right)\xi^{2}\left(1-\epsilon_{n}\right)\right]$ by $\ln\left[1+\left(\frac{n}{2}-1\right)\xi^{2}\left(1+\epsilon_{n}\right)\right]$ in Equation (A9); upperbounding $\ln r-r+1\leq -{\left(r-1\right)}^{2}/2+{\left|r-1\right|}^{3}/3$; and by replacing $e^{-\left(\frac{n}{2}-1\right)\frac{2\epsilon_{n}^{3}}{3}}$ by $e^{\left(\frac{n}{2}-1\right)\frac{\epsilon_{n}^{3}}{3}}$ in Equation (A11). This implies that $I_{2n}∼\ln\left(\frac{n}{2}\;\xi^{2}\right)$, which in turn implies by virtue of Equations (A5) and (A8) that

(A13) $$\frac{n+1}{2}\;I_{n}∼\frac{n+1}{2}\ln\left(\frac{n}{2}\;\xi^{2}\;\right)+o\left(\ln n\right).$$

Using Stirling’s approximation in addition to Equations (A13), Equation (A1) gives

$$\begin{array}{cc} D(p_{G}∥p_{C}) & =-\frac{n}{2}\ln\left(\pi e\xi^{2}\right)-\ln\frac{\Gamma \left(\frac{n+1}{2}\right)}{\pi^{\frac{n+1}{2}}}+\frac{n+1}{2}\;I_{n}\\ \; & ∼-\frac{n}{2}\ln\left(\pi e\xi^{2}\right)-\frac{n-1}{2}\ln\left(\frac{n-1}{2}\right)+\frac{n-1}{2}-\frac{1}{2}\ln\left(\frac{n-1}{2}\right)+\frac{n+1}{2}\ln\pi \\ \; & \;+\frac{n+1}{2}\ln\left(\frac{n}{2}\;\xi^{2}\;\right)+o\left(\ln n\right)\\ \; & ∼\frac{1}{2}\ln n. \end{array}$$

## Appendix B. Examples of Correlated Hypotheses

### Appendix B.1. Example 1

Let 0<α<1. Consider the following binary hypothesis testing problem

$$\begin{array}{c} \left\{\begin{array}{cc} H_{G}: & {\left\{X_{i}\right\}}_{i=1}^{n}\;\mathrm{IID}\;∼B\left(\frac{1}{2}\right)\;\\ H_{C}: & \left\{\begin{array}{cc} X_{1}∼B\left(\frac{1}{2}\right)\;\;\&\;\;X_{1}=\cdots =X_{n} & w.p.\;\;\alpha \\ {\left\{X_{i}\right\}}_{i=1}^{n}\;\mathrm{IID}\;∼B\left(\frac{1}{2}\right) & w.p.\;\;(1-\alpha ), \end{array}\right. \end{array}\right. \end{array}$$

where B(p) is the Bernoulli variable with parameter *p*. It can be readily derived that the Maximum Likelihood (ML) device—optimal in a Bayesian setting with equal a priori—decides on $H_{C}$ whenever all $X_{i}$s are equal and on $H_{G}$ otherwise. Computing $P_{C|G}$:

$$\begin{array}{c} P_{C|G}=\mathrm{Pr}\left(\hat{H}=H_{C}|H_{G}\right)=\mathrm{Pr}\left(\left(\underset{n\;\;\mathrm{times}}{\underset{︸}{0,0,\cdots ,0}}\right)\cup \left(\underset{n\;\;\mathrm{times}}{\underset{︸}{1,1,\cdots ,1}}\right)|H_{G}\right)={\left(\frac{1}{2}\right)}^{n-1}, \end{array}$$

which is exponentially decaying in *n*. As for $\left(1-P_{G|C}\right)$:

$$\begin{array}{cc} \left(1-P_{G|C}\right)=\;\mathrm{Pr}\left(\hat{H}=H_{C}|H_{C}\right)=\; & \mathrm{Pr}\left(\left(\underset{n\;\;\mathrm{times}}{\underset{︸}{0,0,\cdots ,0}}\right)\cup \left(\underset{n\;\;\mathrm{times}}{\underset{︸}{1,1,\cdots ,1}}\right)|H_{C}\right)\\ =\; & 2\left(\frac{\alpha }{2}+\left(1-\alpha \right){\left(\frac{1}{2}\right)}^{n}\right), \end{array}$$

which implies that

$$P_{G|C}=\left(1-\alpha \right)\left(1-{\left(\frac{1}{2}\right)}^{n-1}\right),$$

converging to a positive constant as *n* goes to infinity and is clearly different in behavior from $P_{C|G}$ at large values of *n*.

### Appendix B.2. Example 2: The Beta-Binomial Distribution

Let

$$\begin{array}{c} \left\{\begin{array}{cc} H_{G}: & {\left\{X_{i}\right\}}_{i=1}^{n}\;\mathrm{IID}\;∼B\left(\frac{1}{2}\right)\;\\ H_{C}: & \mathrm{BB}(n,1,1),\;\mathrm{correlated} \end{array}\right. \end{array}$$

where BB(n,α,β) is the Beta-Binomial distribution with parameters n∈N, and α,β>0 [23,24]. Under the BB(n,α,β) distribution, the probability of having *i* zeros among length-*n* sequences is equal to:

(A14) $$b_{n}\left(i\right)=\left(\begin{array}{c} n\\ i \end{array}\right)\frac{B(\alpha +i,n+\beta -i)}{B(\alpha ,\beta )},$$

where B(α,β) is the Beta function given in terms of the Gamma function as follows:

$$B\left(\alpha ,\beta \right)=\frac{\Gamma (\alpha )\Gamma (\beta )}{\Gamma (\alpha +\beta )}.$$

In our case, α=β=1, and $b_{n}\left(i\right)$ evaluates to:

(A15) $$\begin{array}{cc} b_{n}\left(i\right) & =\left(\begin{array}{c} n\\ i \end{array}\right)\frac{B(1+i,n+1-i)}{B(1,1)}=\left(\begin{array}{c} n\\ i \end{array}\right)\frac{i!\;(n-i)!}{(n+1)!}\\ \; & =\frac{1}{n+1},\;0\leq i\leq n. \end{array}$$

Under equal a priori probabilities $p_{0}=p_{1}=\frac{1}{2}$, the MAP detector is an ML one and will choose hypothesis $H_{C}$ whenever the length-*n* sequence is more probable under $H_{C}$ than under $H_{G}$. Let *i* be the number of zeros in a length-*n* sequence. Then, $\hat{H}=H_{C}$ whenever:

(A16) $$\begin{array}{c} \frac{B\left(1+i,n+1-i\right)}{B\left(1,1\right)}\geq {\left(\frac{1}{2}\right)}^{n}\Leftrightarrow \frac{i!\;(n-i)!}{(n+1)!}\geq {\left(\frac{1}{2}\right)}^{n}\Leftrightarrow \left(\begin{array}{c} n\\ i \end{array}\right)\leq \frac{2^{n}}{n+1}. \end{array}$$

We note that whenever there exists an index *i* satisfying Equation (A16), then (n−i) will also satisfy Equation (A16). For the range $\lceil \frac{n}{2}\rceil \leq i\leq n$, let $i_{0}\left(n\right)$ be the smallest integer for which Equation (A16) is satisfied. For large *n*, we claim that:

(A17) $$\frac{i_{0}\left(n\right)-\frac{n}{2}}{\sqrt{n}}∼c\;\sqrt{\ln n}$$

for $c=\frac{1}{2}$. Define $i^{*}\left(n\right)=\left\lceil \frac{n}{2}+c\sqrt{n\ln n}\right\rceil$. Since, for any c>0, we have $\left|\frac{n}{2}-i^{*}(n\right)|=o\left(n^{\frac{2}{3}}\right)$, then using Stirling’s approximation for the binomial coefficient for large *n* and large *i* [25], we have:

(A18) $$\begin{array}{cc} \left(\begin{array}{c} n\\ i^{*}\left(n\right) \end{array}\right) & ∼\frac{2^{n}}{\sqrt{\frac{\pi }{2}\;n}}e^{-\frac{{\left(n-2i^{*}\left(n\right)\right)}^{2}}{2n}}∼\sqrt{\frac{2}{\pi }}\frac{2^{n}}{n^{\frac{1}{2}+2c^{2}}}. \end{array}$$

Equation (A18) violates condition Equation (A16) whenever $c<\frac{1}{2}$ and satisfies it whenever $c\geq \frac{1}{2}$, thus proving our claim. For large *n*, we have

$$\begin{array}{cc} P_{C|G} & =\mathrm{Pr}\left(\hat{H}=H_{C}|H_{G}\right)={\left(\frac{1}{2}\right)}^{n}2\sum_{i_{0}\left(n\right)}^{n}\left(\begin{array}{c} n\\ i \end{array}\right)={\left(\frac{1}{2}\right)}^{n}\left(2^{n}-\sum_{n-i_{0}\left(n\right)+1}^{i_{0}\left(n\right)-1}\left(\begin{array}{c} n\\ i \end{array}\right)\right) \end{array}$$

(A19) $$\begin{array}{cc} & \;∼1-\sqrt{\frac{2}{\pi }}\frac{1}{\sqrt{n}}\sum_{n-i_{0}\left(n\right)+1}^{i_{0}\left(n\right)-1}e^{-\frac{{\left(n-2i\right)}^{2}}{2n}} \end{array}$$

(A20) $$\begin{array}{cc} & \;∼1-\frac{2}{\sqrt{n}}\frac{1}{\sqrt{2\pi }}\int_{n-i_{0}\left(n\right)+1}^{i_{0}\left(n\right)-1}e^{-\frac{{\left(n-2t\right)}^{2}}{2n}}\;dt \end{array}$$

(A21) $$\begin{array}{cc} & \;=1-\left[Q\left(\frac{2i_{0}\left(n\right)-n-2}{\sqrt{n}}\right)-Q\left(\frac{-2i_{0}\left(n\right)+n+2}{\sqrt{n}}\right)\right]\\ & \;=2Q\left(\frac{2i_{0}\left(n\right)-n-2}{\sqrt{n}}\right) \end{array}$$

(A22) $$\begin{array}{cc} & \;∼\sqrt{\frac{2}{\pi }}\frac{e^{-\frac{{\left(2i_{0}\left(n\right)-n-2\right)}^{2}}{2n}}}{\frac{2i_{0}\left(n\right)-n-2}{\sqrt{n}}} \end{array}$$

(A23) $$\begin{array}{cc} & \;∼\sqrt{\frac{2}{\pi }}\frac{1}{\sqrt{n\ln n}} \end{array}$$

where we used Stirling’s approximation to write Equation (A19). Equation (A20) is valid since $e^{-\frac{{\left(n-2t\right)}^{2}}{2n}}∼\frac{1}{\sqrt{n}}$ is convex for $n-i_{0}\left(n\right)+1\leq t\leq i_{0}\left(n\right)-1$, thus implying:

$$\begin{array}{c} \int_{n-i_{0}\left(n\right)+1}^{i_{0}\left(n\right)-1}e^{-\frac{{\left(n-2t\right)}^{2}}{2n}}\;dt\leq \sum_{n-i_{0}\left(n\right)+1}^{i_{0}\left(n\right)-1}e^{-\frac{{\left(n-2i\right)}^{2}}{2n}}\leq \int_{n-i_{0}\left(n\right)}^{i_{0}\left(n\right)-1}e^{-\frac{{\left(n-2t\right)}^{2}}{2n}}\;dt, \end{array}$$

with both the upper and lower bounds having the same asymptotic behavior. Equation (A21) is due to the change of variable $u=\frac{2t-n}{\sqrt{n}}$, and Equation (A22) is justified by using the approximation in Equation (25). Finally, Equation (A23) is justified by Equation (A17). As for $P_{G|C}$, we have

$$\begin{array}{cc} P_{G|C}=\; & \mathrm{Pr}\left(\hat{H}=H_{G}|H_{C}\right)=\sum_{n-i_{0}\left(n\right)+1}^{i_{0}\left(n\right)-1}\;b_{n}\left(i\right)=\frac{1}{n+1}\;\left(2i_{0}\left(n\right)-n-2\right)∼\sqrt{\frac{\ln n}{n}}, \end{array}$$

where $b_{n}\left(i\right)$ is given in Equation (A15) and where we used Equation (A17) to write the last equation. Once more, $P_{C|G}$ and $P_{G|C}$ exhibit different asymptotic behaviors.

### Appendix B.3. Example 3: Continuous Laws

Given n∈N, n≥3, consider the following PDF over ${\left[0,1\right]}^{n}$:

$$\begin{array}{c} p_{n}\left(x^{n}\right)=\left\{\begin{array}{cc} n-\sqrt{n}, & \mathrm{if}\;∥x^{n}{∥}_{\infty }\leq n^{-1/n},\;\\ \frac{\sqrt{n}}{n-1}, & \mathrm{otherwise}, \end{array}\right. \end{array}$$

where $∥x^{n}{∥}_{\infty }=\max_{i}\left|x_{i}\right|$. It can be easily verified that $p_{n}$ is a valid PDF by noting that the volume of the set $\left\{x^{n}:∥x^{n}{∥}_{\infty }\leq n^{-1/n}\right\}$ is $\frac{1}{n}$.

Now, consider the following hypothesis testing problem:

$$\begin{array}{c} \left\{\begin{array}{c} H_{G}:{\left\{X\right\}}_{i=1}^{n}\;\mathrm{IID}\;∼U\left[0,1\right],\;\\ H_{C}:X^{n}∼p_{n}\left(\cdot \right), \end{array}\right. \end{array}$$

where U[0,1] is the uniform law on the interval [0,1].

Assuming a priori uniform over $H_{G}$ and $H_{C}$, the optimal ML decision rule yields

$$\begin{array}{c} {∥x^{n}∥}_{\infty }\underset{H_{G}}{\overset{H_{C}}{⋚}}n^{-1/n}. \end{array}$$

As such,

$$\begin{array}{cc} P_{C|G} & =\mathrm{Pr}\left(\hat{H}=H_{C}|H_{G}\right)=\mathrm{Pr}\left({∥X^{n}∥}_{\infty }\leq n^{-1/n}|H_{G}\right)=\frac{1}{n}. \end{array}$$

On the other hand,

$$\begin{array}{cc} P_{G|C} & =\mathrm{Pr}\left(\hat{H}=H_{G}|H_{C}\right)=\mathrm{Pr}(∥X^{n}{∥}_{\infty }>n^{-1/n}\left|H_{C}\right)=\int_{\left\{∥x^{n}{∥}_{\infty }>n^{-1/n}\right\}}\frac{\sqrt{n}}{n-1}\;dx^{n}\\ \; & =\frac{\sqrt{n}}{n-1}\left(1-\int_{\left\{∥x^{n}{∥}_{\infty }\leq n^{-1/n}\right\}}dx^{n}\right)=\frac{\sqrt{n}}{n-1}\left(1-\frac{1}{n}\right)=\frac{1}{\sqrt{n}}, \end{array}$$

and the asymptotic behavior is clearly different from that of $P_{C|G}$.

## Appendix C. Asymptotics of $P_{G|C}$

In this appendix, we show the following technical result

(A24) $$\begin{array}{c} P_{G|C}\geq \frac{1}{\sqrt{\pi }}\frac{\Gamma \;\left(\frac{n+1}{2}\right)}{\Gamma \;\left(\frac{n}{2}\right)}\sqrt{\frac{2}{n+1}}\;\left(\Gamma \;\left(\frac{1}{2},\frac{n+1}{2r_{2}^{2}}\right)\;-\;\Gamma \;\left(\frac{1}{2},\frac{n+1}{2r_{1}^{2}}\right)\right). \end{array}$$

Furthermore, under Regime (R-i), we prove that

(A25) $$\begin{array}{c} P_{G|C}∼\frac{1}{\sqrt{\pi }}\frac{\Gamma \;\left(\frac{n+1}{2}\right)}{\Gamma \;\left(\frac{n}{2}\right)}\sqrt{\frac{2}{n+1}}\;\left(\Gamma \;\left(\frac{1}{2},\frac{n+1}{2r_{2}^{2}}\right)\;-\;\Gamma \;\left(\frac{1}{2},\frac{n+1}{2r_{1}^{2}}\right)\right). \end{array}$$

To show Equation (A24), we use the expression of $P_{G|C}$ as given by Equation (11) and the fact that ${\left(1+u\right)}^{-\frac{n+1}{2}}\geq e^{-\frac{n+1}{2}u}$ for u≥0, and we write:

$$\begin{array}{cc} P_{G|C} & =\frac{1}{\sqrt{\pi }}\frac{\Gamma \;\left(\frac{n+1}{2}\right)}{\Gamma \;\left(\frac{n}{2}\right)}\int_{\frac{1}{r_{2}^{2}}}^{\frac{1}{r_{1}^{2}}}{\left(1+u\right)}^{-\frac{n+1}{2}}u^{-\frac{1}{2}}\;du\geq \frac{1}{\sqrt{\pi }}\frac{\Gamma \;\left(\frac{n+1}{2}\right)}{\Gamma \;\left(\frac{n}{2}\right)}\int_{\frac{1}{r_{2}^{2}}}^{\frac{1}{r_{1}^{2}}}e^{-\frac{n+1}{2}u}u^{-\frac{1}{2}}\;du\\ \; & =\frac{1}{\sqrt{\pi }}\frac{\Gamma \;\left(\frac{n+1}{2}\right)}{\Gamma \;\left(\frac{n}{2}\right)}\sqrt{\frac{2}{n+1}}\;\left(\Gamma \;\left(\frac{1}{2},\frac{n+1}{2r_{2}^{2}}\right)-\Gamma \;\left(\frac{1}{2},\frac{n+1}{2r_{1}^{2}}\right)\right) \end{array}$$

where the last equation is due to the definition of the upper incomplete Gamma function. To further show Equation (A25), we note that under Regime (R-i), the following holds:

$$\begin{array}{cc} \lim_{n\to \infty }\frac{\frac{1}{\sqrt{\pi }}\frac{\Gamma \left(\frac{n+1}{2}\right)}{\Gamma \left(\frac{n}{2}\right)}\int_{\frac{1}{r_{2}^{2}}}^{\frac{1}{r_{1}^{2}}}{\left(1+u\right)}^{-\frac{n+1}{2}}u^{-\frac{1}{2}}\;du}{\frac{1}{\sqrt{\pi }}\frac{\Gamma \left(\frac{n+1}{2}\right)}{\Gamma \left(\frac{n}{2}\right)}\int_{\frac{1}{r_{2}^{2}}}^{\frac{1}{r_{1}^{2}}}e^{-\frac{n+1}{2}u}u^{-\frac{1}{2}}\;du}\; & =\lim_{n\to \infty }\frac{\int_{\frac{1}{r_{2}^{2}}}^{\frac{1}{r_{1}^{2}}}e^{-\frac{n+1}{2}\left[\ln(1+u)-u\right]}e^{-\frac{n+1}{2}u}u^{-\frac{1}{2}}\;du}{\int_{\frac{1}{r_{2}^{2}}}^{\frac{1}{r_{1}^{2}}}e^{-\frac{n+1}{2}u}u^{-\frac{1}{2}}\;du} \end{array}$$

(A26) $$\begin{array}{cc} & \;\;\;\;\;\;\;\;\;\;\;\;\;\;\;\;\;\leq \lim_{n\to \infty }e^{-\frac{n+1}{2}\left[\ln\left(1+\frac{1}{r_{1}^{2}}\right)-\frac{1}{r_{1}^{2}}\right]}\\ & \;\;\;\;\;\;\;\;\;\;\;\;\;\;\;\;\;=\left[\lim_{n\to \infty }e^{-\frac{n+1}{2}\ln\left(1+\frac{1}{r_{1}^{2}}\right)}\right]\left[\lim_{n\to \infty }e^{\frac{n+1}{2}\frac{1}{r_{1}^{2}}}\right] \end{array}$$

(A27) $$\begin{array}{cc} & \;\;\;\;\;\;\;=e^{-\frac{1}{\xi^{2}}}e^{\frac{1}{\xi^{2}}}=1, \end{array}$$

where in order to write Equation (A27), we used the fact that $r_{1}^{2}$ behaves according to Equation (13) under Regime (R-i). Equation (A26) holds true since ln(1+u)−u is non-increasing for u≥0.

## Appendix D. Numerical Simulations

We compute the performance of the LRT defined in Equation (1) by evaluating the different quantities studied in Theorems 1 and Equation (2). For the results presented hereafter, we use $\xi =\sqrt{2}$ and dimension n∈[11,340]. Figure A1 depicts the behavior of the Bayesian probability of error $P_{e}$ function of *n* for different values of the a priori. Figure A1 shows a rather fast convergence of the approximate expression presented in Theorem 1 to the numerically evaluated $P_{e}$ (3). In Figure A2, we consider Case 1 in Theorem 2 and plot the numerically evaluated error of the second kind $P_{G|C}$ versus *n*, in addition to the approximate expression for different values of ε. Again, the fast convergence of the approximation is observed. Finally, we depict in Figure A3$-\ln P_{C|G}$ as a function of *n*. A linear behavior is noticed, as predicted by the result of Case 2 in Theorem 2.
